# The educational impact of medical students’ participation in a short-term health expedition: The Iguape one health experience

**DOI:** 10.1016/j.clinsp.2025.100601

**Published:** 2025-02-12

**Authors:** Marcelo Arruda Candido, Vinicius Venturini, Matheus Polly, Matheus Belloni Torsani, Alexandre Sizilio, João Mitsuji Sakô, Wen-Jan Tuan, Robert Patrick Lennon, Anna Sara Shafferman Levin, Iolanda de Fátima Lopes Calvo Tibério

**Affiliations:** a Center for the Development of Medical Education, Faculdade de Medicina da Universidade de São Paulo (FMUSP), São Paulo, SP, Brazil; b Division of Infectious Diseases, Hospital das Clínicas, Faculdade de Medicina da Universidade de São Paulo (FMUSP), São Paulo, SP, Brazil; c Faculdade Israelita de Ciências da Saúde Albert Einstein (FICSAE), São Paulo, SP, Brazil; d Municipal Department of Health, Iguape, SP, Brazil; e Department of Family and Community Medicine, College of Medicine, Pennsylvania State University, Hershey, PA, USA; f Department of Infectiology and Tropical Medicine, Faculdade de Medicina da Universidade de São Paulo (FMUSP), São Paulo, SP, Brazil; g Department of Internal Medicine, Faculdade de Medicina da Universidade de São Paulo (FMUSP), São Paulo, SP, Brazil

**Keywords:** Health expeditions, Educational evaluation, Medical students, Professional Orientation, Public Health System

## Abstract

**Objectives:**

The primary goal of this study is to evaluate the educational effects of medical students' participation in a health expedition project. Specific objectives include assessing students' perceptions of the educational value of the program, measuring gains in clinical knowledge and skills, evaluating behavioral changes, professional orientation, and students' willingness to work within the public health system (SUS) after graduation.

**Methods:**

A quantitative methodology was employed, following Kirkpatrick's four-level evaluation model. Questionnaires were administered before and after participation in the expedition to assess students' reactions, learning, and empathy. The sample comprised all 15 students who participated in the expedition, with data analysis performed using R software.

**Results:**

The analysis highlighted that the expedition experience significantly impacted student's career trajectories. Approximately 80 % of participants reported that the expedition positively influenced their career decisions, and 60 % expressed interest in working within the SUS. These findings suggest that while expeditions may not yield quantifiable gains in clinical competence in the short term, they can be catalysts in forming physicians oriented towards public service and social sensitivity.

**Conclusions:**

Health expeditions like the one conducted may not offer significant improvements in measurable clinical competencies in the short term but have a substantial effect on students' career decisions, aligning with public health needs and reinforcing commitment to service in vulnerable contexts. This influence suggests that practical experiences in community settings play a crucial role in shaping medical students' professional aspirations, emphasizing the importance of such programs in medical education aimed at social responsibility and health equity.

## Highlights

• Educational value of health expeditions assessed via surveys.
• No significant clinical or empathy gains observed.
• 80 % reported positive career influence from participation.
• 60 % intend to work within the public health system.
• Practical experiences deemed crucial for shaping careers.

## Introduction

The high interest among medical students in participating in medical expeditions reflects a significant trend in medical education, where such endeavors are recognized not just for their immediate health impacts but also for their educational potential. These expeditions, however, come with substantial logistical, financial, and infrastructural challenges that institutions need to manage effectively. Successful trips required considerable organizational effort from both students and faculty, emphasizing the need for strong institutional support to maximize the educational value of such initiatives.^1^

The motivations of medical students to participate in medical missions are multifaceted, primarily driven by the desire for enhanced clinical experience, exposure to diverse medical conditions, and a profound commitment to providing service to underserved populations. Gishen and Thaller (2015) highlight that students are often motivated by the opportunity to apply their medical knowledge in real-world settings, which not only enriches their understanding of diverse medical practices but also helps them develop essential clinical skills that are difficult to replicate in a traditional classroom setting.^2^ Furthermore, according to Rovers et al. (2014), students also participate with the intention of gaining a deeper understanding of global health disparities and developing a broader cultural competence, which are increasingly recognized as vital components of medical education.^3^ These expeditions provide a platform for students to engage directly with patients in resource-limited settings, fostering a sense of empathy and a long-lasting commitment to addressing healthcare inequities.

Extensive literature highlights both the advantages and complexities associated with medical missions. Medical expeditions, while beneficial in providing direct relief to underserved areas, have faced criticism for potentially fostering dependency and not addressing the underlying health issues in host communities.^4^^,^^5^ However, studies have shown that such experiences can significantly enhance medical students' clinical skills, cultural competence, and understanding of global health disparities.^6^^,^^7^ Additionally, integrating a research component into these missions has been suggested as a means to extend their impact, creating sustainable health benefits that go beyond the duration of the trips.^4^

This study aims to evaluate the educational impacts of participation by Brazilian medical students in an interprofessional health expedition to Iguape ‒ a region characterized by limited medical services and diverse traditional communities. It seeks to determine the immediate and long-term educational outcomes of such experiences and to understand how they influence students' career trajectories. Through this research, the authors aim to contribute to the discourse on how medical expeditions can be structured to provide maximum educational value.

## Material and methods

### One health service trip to Iguape

The Universidade de São Paulo, in partnership with the Iguape municipality and Pennsylvania State University, organized a week-long interprofessional health expedition to the town of Iguape. The expedition aimed to provide specialized health services to the population of Iguape by evaluating patients with complex clinical conditions that were previously triaged by the local primary care physicians, offering training to local health professionals, conducting health education activities with the community, and carrying out scientific research under the framework of One Health, focusing on the interaction between health and the environment.

Iguape is a coastal town in the state of São Paulo with 29,115 inhabitants,^8^ and with a structured primary health care system but underserved by medium and high complexity medical services. It is characterized by a low-income region and comprises a vast rural area and several traditional communities, such as quilombolas and riverside dwellers. These factors justified the selection of this location for the expedition.

The expedition was designed as an interprofessional initiative, driven by the health needs of the community. In addition to medical students from the University of São Paulo Medical School, the team included specialists and residents from the USP in five different medical specialties (infectious diseases, ophthalmology, dermatology, internal medicine, and cardiovascular surgery), nurses and nursing technicians, psychologists, physical educators, environmental agents, and a support team. All clinical cases could be promptly discussed over the phone with experienced specialists in each field who were stationed at USP's tertiary care hospital (Hospital das Clínicas) and available during the entire expedition.

The students actively participated in all on-site activities of the expedition, including organizing the work environment, guiding and screening patients, conducting health promotion activities, providing medical care in the available specialties, discussing cases, and performing additional diagnostic tests. Travel was funded by USP and stay was funded by the Iguape municipality.

### Study design and participants

A total of 15 medical students from the University of São Paulo, among 50 candidates, were selected through a public announcement to participate in the expedition. Eligibility was restricted to students in the last three years of their six-year medical degree, a period characterized by increased clinical practice, who had not previously participated in any voluntary expeditions. The selection process was based on academic merit, and all chosen students were invited to participate in the study.

To evaluate the educational effects of the student's participation in the medical trip, the authors adapted Kirkpatrick's four-level evaluation model into two questionnaires, administered before (Survey 1) and after (Survey 2) the expedition. Kirkpatrick's model categorizes the outcomes of an educational program into four hierarchical levels: (1) Learner satisfaction or reaction to the program; (2) Measures of learning attributed to the program; (3) Changes in learner behavior in the context for which they are being trained; and (4) The program's final results in its larger context, as outlined in the AMEE Guide no 67.^9^

### Data collection and analysis

To assess participant satisfaction with the program, the authors employed the Net Promoter Score (NPS), which measures the likelihood of respondents recommending the experience to a colleague. The NPS was calculated based on the proportion of promoters (those who gave ratings of 9 or 10), passives (ratings of 7 and 8), and detractors (ratings from 0 to 6) among the respondents.

Given that the students were immersed in a practical setting, the authors applied the adapted Calgary questionnaire for self-assessment of clinical skills to measure potential practical learning outcomes from participating in the program.^10^ The questionnaire consists of 12 items and is structured on a 5-point Likert scale, which was previously validated and utilized by other researchers in Brazilian Portuguese.^11^ For comparative purposes, all questions were administered in both Survey 1 and Survey 2.

To measure behavioral changes that could be attributed to the program, the authors administered the Toronto Empathy Questionnaire (TEQ) before and after the trip. The TEQ is a Portuguese-validated self-report questionnaire with 16 items on a 5-point Likert scale designed to assess empathy.^12^^,^^13^ This tool was chosen based on the understanding that students would be exposed to a social and cultural context different from that of their university setting, which could potentially influence their empathy levels.

Finally, to evaluate the eventual final outcomes for participants resulting from the program, beyond healthcare delivery and local health impacts, the authors sought to determine whether participation influenced crucial future choices of the participants, such as their intended medical careers. The authors analyzed participants' responses to identify any changes or influences on their professional decision-making.

All statistical data analyses were performed using R version 4.03. The authors adopted a 95 % Confidence Interval and a significance level of 5 %. Paired *t*-tests were applied to compare participants’ responses before and after the trip, analyzing whether there were statistically significant changes in the perception of skills and behaviors. The study was appreciated and approved by the Commission of Ethics in Research of the University of São Paulo Medical School.

## Results

### Expedition outcomes

During the expedition, four training sessions were conducted for community health agents, covering topics such as vaccination, environmental health, tuberculosis, and cardiovascular prevention, reaching approximately 190 participants, both onsite and remotely. Concurrently, about 60 healthcare professionals, including physicians, nurses, and pharmacists, underwent advanced training in emergency procedures, cardiac arrhythmias, and the management of acute pulmonary edema, topics that were preselected by the local professionals themselves. In terms of healthcare delivery, the expedition documented the screening and treatment of 739 patients, which included consultations in general medicine, 116 in dermatology, 344 in ophthalmology, and 103 in vascular surgery, leading to several referrals for specialized care.

### Participant profile

The study involved a diverse group of medical students. Of the participants, 6 (40 %) were fourth-year students, 4 (27 %) were fifth-year students, and 33 % (*n* = 5) were in their sixth year. In terms of demographic distribution, 10 (67 %) of the participants identified as White, 3 (20 %) as Black or Brown, and 4 (13.3 %) as Asian. Gender distribution was relatively balanced with 9 (60 %) female and 6 (40 %) male participants, indicative of the current trends in medical education enrollment.

### Kirkpatrick's level 1: reaction

The educational impact of the expedition was highly rated by the participants, as evidenced by the Net Promoter Score. The overall NPS reached 100 %, with 14 participants rating the experience as a perfect ten and one participant giving a score of nine, categorizing all respondents as promoters.

### Kirkpatrick's level 2: learning

The evaluation of learning outcomes using the adapted Calgary questionnaire aimed to assess changes in clinical skills before and after participation in the medical expedition. Statistical analysis was performed using paired *t*-tests for each item to discern significant learning enhancements among participants. The overall analysis of the questionnaire items, as summarized in Table 1, revealed minimal statistically significant changes in the clinical skills of participants.

**Table 1:** Paired *t*-test results for pre- and post-expedition clinical skills self-assessment.

Items	Pre Mean	Post Mean	Cohen's d	p
1. Identify problems the patient's wishes to address	4.5	4.4	−0.2	0.433
2. Use concise, easily understood, jargon-free language	4.7	4.3	−0.6	0.028^a^
3. Structure interview in logical sequence	4.1	3.9	−0.1	0.582
4. Attend to time keeping, and keeping interview on task	3.5	3.8	0.3	0.334
5. Use appropriate non-verbal behavior	4.6	4.3	−0.3	0.217
6. Provide support: express concern, understanding, and willingness to help	4.7	4.5	−0.4	0.104
7. Share thought and reflection with the patient	4.1	3.9	−0.2	0.546
8. Clarify patient's prior knowledge and wish for information	4.3	4.3	−0.1	0.774
9. Check patient's understanding	4.5	4.5	0.0	1.000
10. Negotiate mutual plan of action	3.7	3.9	0.4	0.164
11. Contract with patient the next steps for patient and physician	3.9	4.1	0.2	0.546
12. Summarise session briefly and clarify plan of care	4.3	4.4	0.1	0.774

a *p* < 0.05 %. This table shows paired *t*-test results for pre- and post-intervention assessments on the Calgary questionnaire using a Likert scale from 1–5 (strongly disagree to strongly agree).

**Fig. 1:**
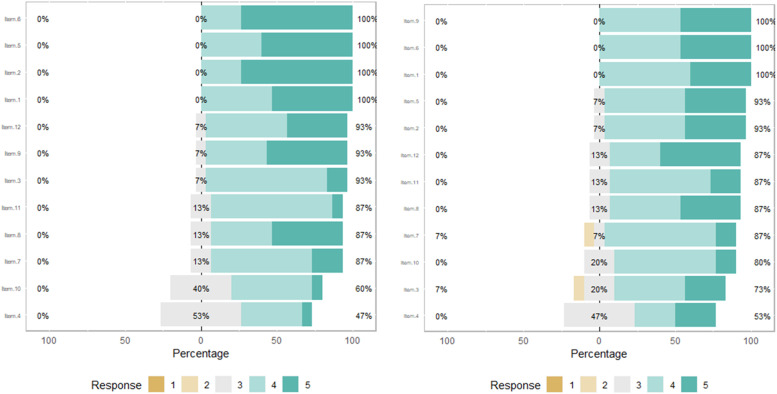
Response distribution of the adapted calgary questionnaire pre- and post-expedition. This graph displays the distribution of responses to the Adapted Calgary questionnaire before (left panel) and after (right panel) the medical expedition. Responses are rated on a 5-point Likert scale where 1 signifies “Strongly Disagree”, and 5 signifies “Strongly Agree”.

The majority of the items, including those related to essential clinical practices showed no significant improvement. Significantly, Item 2, which assessed the effectiveness of communication in practice, experienced an unexpected decrease in mean scores from 4.7 pre-intervention to 4.3 post-intervention (*p* = 0.028). Fig. 1 displays the results from the adapted Calgary questionnaire, showing minimal changes in clinical skills before and after the expedition.

### Kirkpatrick's level 3: behavior

**Fig. 2:**
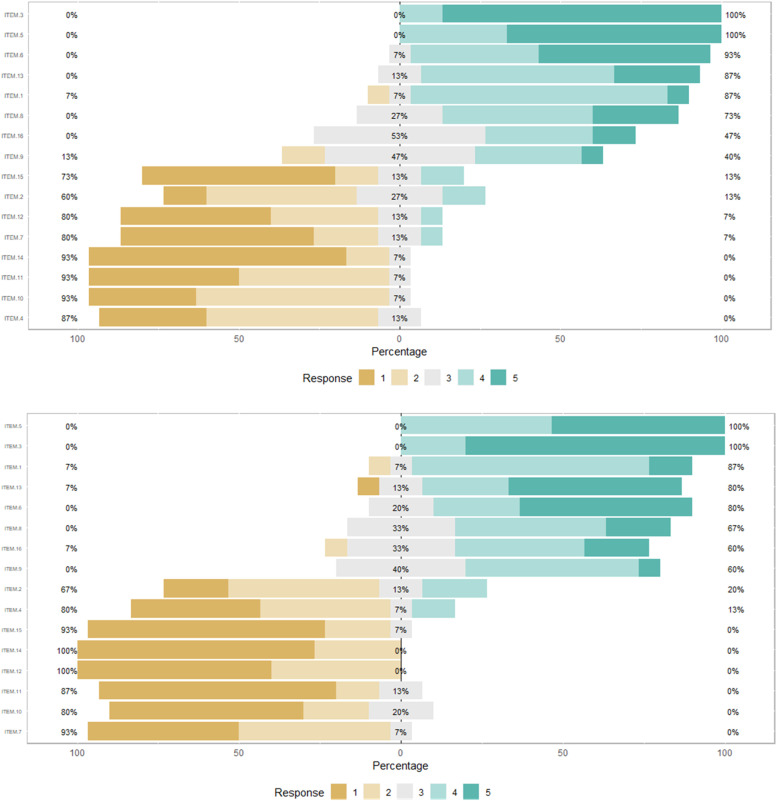
Response Distribution of the Toronto Empathy Questionnaire Pre- and Post-Expedition. This graph displays the distribution of responses to the Toronto Empathy Questionnaire before (left panel) and after (right panel) the medical expedition. Responses are rated on a 5-point Likert scale where 1 signifies “Never”, and 5 signifies “Always”.

The authors employed the Toronto Empathy Questionnaire (TEQ) to evaluate changes in empathy as a behavioral metric, following participation in the medical expedition. This aspect of the evaluation was aimed at assessing whether the experiential learning environment influenced the empathic responses of the medical students toward patients. The TEQ scores were analyzed using paired *t*-tests to compare pre-expedition and post-expedition responses, as shown in Table 2. Additionally, Fig. 2 presents the findings from the Toronto Empathy Questionnaire (TEQ), illustrating the stability of empathy scores across all items.

**Table 2:** Paired *t*-test results for pre- and post-expedition empathy assessment.

Items	Pre Mean	Post Mean	Cohen's d	p
1. When someone else is feeling excited, I tend to get excited too	3.9	3.9	0.08	0.774
2. Other people's misfortunes do not disturb me a great deal	2.4	2.3	−0.05	0.860
3. It upsets me to see someone being treated disrespectfully	4.9	4.8	−0.15	0.582
4. I remain unaffected when someone close to me is happy	1.8	1.9	0.10	0.709
5. I enjoy making other people feel better	4.7	4.5	−0.21	0.433
6. I have tender, concerned feelings for people less fortunate than me	4.5	4.3	−0.13	0.634
7. When a friend starts to talk about his/her problems, I try to steer the conversation towards something else	1.7	1.6	−0.05	0.836
8. I can tell when others are sad even when they do not say anything	4.0	3.9	−0.13	0.634
9. I find that I am “in tune” with other people's moods	3.3	3.7	0.32	0.238
10. I do not feel sympathy for people who cause their own serious illnesses	1.7	1.6	−0.16	0.546
11. I become irritated when someone cries	1.6	1.4	−0.21	0.424
12. I am not really interested in how other people feel	1.8	1.4	−0.48	0.082
13. I get a strong urge to help when I see someone who is upset	4.1	4.2	0.06	0.827
14. When I see someone being treated unfairly, I do not feel very much pity for them	1.3	1.3	0.00	1.000
15. I find it silly for people to cry out of happiness	1.8	1.3	−0.39	0.150
16. When I see someone being taken advantage of, I feel kind of protective towards him/her	3.6	3.7	0.11	0.685

This table shows paired *t*-test results for pre- and post-intervention assessments on the Toronto Empathy Questionnaire using a Likert scale from 1–5 (Never to Always).

### Kirkpatrick's level 4: results

The fourth level of Kirkpatrick's model assesses the long-term impact of educational interventions, focusing particularly on how such experiences influence career planning and professional choices. In this study, the expedition was evaluated for its potential effects on medical students' future career decisions.

The majority of participants acknowledged that the experience had influenced their career choices: 8 students (53 %) strongly agreed, and 4 students (27 %) agreed partially that the expedition impacted their decision regarding their future medical specialty. However, 2 students (13 %) disagreed completely, and 1 student (7 %) remained neutral, indicating that the expedition did not sway their career plans. This feedback highlights a significant, though not universal, influence of the practical experience gained during the expedition on career orientation.

Regarding the choice of working environment, particularly in relation to the public health system, a notable preference emerged among the participants for serving in the public sector. Sixty percent (9 students) expressed a preference to work primarily with patients in the Brazilian Public Health System (Sistema Único de Saúde – SUS), and an additional 1 student wished to work exclusively with these patients. Conversely, 5 students (33 %) planned to engage minimally with SUS-dependent patients, indicating a preference for private practice or mixed settings.

## Discussion

The participant profile in this study, despite comprising only a small number (*n* = 15) relative to the entire student body at the medical school, presents a relatively representative cross-section in terms of gender and racial demographics. The participants were exclusively students who had already received formal training in basic clinical skills, which is critical for understanding the context of their responses and experiences. Importantly, about over half of these students were in their clinical-surgical internship phase (fifth and sixth years), a period marked by intense and daily practical training in a service environment.

The high number of applications for participation in the project (50), coupled with the participants' expressed satisfaction, as evidenced by the Net Promoter Score of 100 %, strongly underscores the findings in the literature regarding students' eagerness to engage in such health service trips.^14^ Students often seek out medical service trips not merely out of an inherent desire to volunteer, but because these opportunities provide a context that aligns closely with their professional and educational goals.^3^ This context-driven motivation aligns well with the observed enthusiasm and satisfaction among participants, who valued the practical application of their clinical skills in a real-world setting. The comprehensive satisfaction demonstrated by every participant categorizing themselves as promoters reflects a significant acknowledgment of the expedition's value, meeting or even exceeding their expectations in terms of educational and professional development.

The findings from the present study on Level 2 of Kirkpatrick's evaluation, specifically focusing on clinical learning outcomes assessed through the adapted Calgary questionnaire, showed minimal statistically significant changes in clinical skills as observed from pre- to post-expedition assessments, which may be partly explained by the small sample and the high initial competency level among participants, limiting the observable impact of the training. Furthermore, the unexpected decrease in the scores for communication effectiveness (Item 2) in this study may reflect the students' reassessment of their communication skills after interacting with patients from culturally and socially diverse backgrounds during the expedition. Such exposure likely heightened their awareness of the complexities involved in effective communication, leading them to perceive that their skills required further enhancement. In contrast, consistent scores across other critical items, such as those assessing procedural skills and patient interaction, underscored a stable but unenhanced skill set post-expedition.

While studies such as those by Vu et al. (2014)[6] and Aziz et al. (2012)[15] have documented the long-term benefits of international medical missions, including enhanced communication skills, and procedural knowledge, these findings present a more nuanced view regarding the efficacy of short-term expeditions as training tools. Vu et al. observed that residents continued to reap benefits in terms of an improved understanding of global health issues well after their missions,^6^ while Aziz et al. reported significant skill enhancements among dental students engaged in overseas clinics.^15^ In contrast, the results did not demonstrate substantial improvements in clinical skills following a short-term medical expedition. This discrepancy suggests that while extended and perhaps repeated engagements in diverse clinical environments may solidify and expand medical knowledge and skills, short-term expeditions might not provide sufficient depth and continuity to serve as effective standalone training tools for health skills enhancement. Furthermore, the clinical skills themselves were not evaluated but the self-perception of the students.

Different authors have shown that international medical missions often result in behavioral growth, such as enhanced cultural sensitivity and social awareness,^6^^,^^7^^,^^15^^,^^16^ these findings did not demonstrate significant changes in behavioral measures such as empathy. Dowell and Merrylees (2009) emphasize the transformative potential of electives in fostering global health perspectives and ethical mindfulness.^16^ Similarly, Vu et al. (2014) document the perception of improved cultural competence among residents post-mission.^6^ However, the present study's lack of significant changes in empathy might reflect the complexity of modifying empathy through short-term interventions, highlighting the need for longer duration or more intensive experiential learning to impact such deeply ingrained personal traits.

These findings imply that while the educational intervention successfully maintained existing levels of empathy, it did not facilitate a statistically significant enhancement or reduction in these skills. The stability of empathy scores across both positive and negative-oriented items suggests that the pre-existing empathetic capacities of participants were robust, potentially due to the high baseline of personal and professional empathy typically found in medical students. However, there may be a selection bias as volunteers for such an activity may already have higher levels of empathy than the entire medical student body.

In Level 4 of Kirkpatrick's model, which assesses the long-term impacts of educational interventions on career planning and professional choices, the present study aligns with findings from prior research indicating that global health experiences can significantly influence medical students' career trajectories, particularly in their specialty choice and practice settings.^17^, ^18^, ^19^, ^20^ Notably, studies by Kaeppler et al. (2021), Bruno et al. (2013), and Chang et al. (2019) have shown that students who engage in global health electives during their training are more likely to choose primary care specialties and express a strong interest in working with underserved populations, both domestically and internationally.^17^, ^18^, ^19^ Additionally, Lee and colleagues (2011) identified that students exposed to rural health experiences are more likely to engage in a career in rural and remote health.^20^

Given the uneven distribution of the healthcare workforce across Brazil, where metropolitan urban areas in various states have more than seven times the number of physicians per capita when compared to rural areas within the same states,^21^ educational strategies that encourage healthcare professionals to pursue careers in rural regions and to work in public health services should be highly valued and meticulously developed.

These findings suggest that the expedition not only provided immediate educational benefits but also played a crucial role in shaping participants' attitudes toward public health and their professional aspirations. The experience seems to have reinforced or possibly initiated an interest in public health careers, a critical insight for medical education programs aiming to address healthcare disparities.

This study faces several limitations. The small sample size of 15 participants and volunteering engagement in the expedition limits the generalizability of the results, and the absence of a control group prevents robust comparisons from being made. Additionally, the short duration of the expedition and immediate post-trip evaluations may not capture the long-term impacts of the educational intervention, while the self-reported nature of the data collection tools could introduce response bias. Finally, the brief duration of the experience may not have been sufficient to effect significant changes in deeply ingrained traits such as empathy and cultural sensitivity, suggesting the need for longer or repeated exposures. Future research could address these limitations by expanding the sample size, including a control group, and using a mix of qualitative and quantitative methods to enhance the reliability and depth of the findings.

A complementary study is currently underway to address some of these limitations by shifting the focus from individual learning outcomes to systemic and structural impacts. This ongoing research evaluates the perspectives of patients and local healthcare professionals involved in similar expeditions conducted by the same institution. By examining how these initiatives influence community health indicators, professional development among local staff, and the overall effectiveness of public health systems, the study aims to provide a more comprehensive understanding of the broader societal benefits and challenges associated with short-term health interventions.

## Conclusion

The significant interest of students and their high satisfaction rates reported post-expedition underscore the appeal and perceived value of medical service trips, resonating with the literature that acknowledges the strong desire among medical students for such experiences. While these findings were not able to determine the efficacy of short-term expeditions in enhancing perceived clinical skills and empathy, they also confirm the influence of these expeditions on career decisions, particularly in the inclination towards careers in the public health system. Further investigation into curriculum design may be warranted to optimize learning outcomes in future programs.

Additionally, given the disproportionate distribution of healthcare professionals across Brazil, the role of educational interventions such as these in encouraging careers in underserved areas is critical. The present findings emphasize the importance of strategically designed medical expeditions that not only aim to provide immediate medical services but also foster long-term commitments among medical students to serve in low-resourced settings. This approach could help address significant disparities in access to healthcare and ensure a more equitable distribution of medical expertise across the country. Future studies should consider larger sample sizes and longer follow-up periods to better understand the enduring impacts of these expeditions on medical education and professional development.

## Declaration

During the preparation of this work, the authors used the tool ChatGPT 4.0® in order to check grammar, spelling, and telling. After using this tool, the authors reviewed and edited the content as needed and took full responsibility for the content of the publication.

## Funding

This project was supported by Fundaão Maria Emília and the University of São Paulo.

## Declaration of competing interest

The authors declare no conflicts of interest.
